# Unified First Order Inertial Element Based Model of Magnetostrictive Hysteresis and Lift-Off Phenomenon

**DOI:** 10.3390/ma12101689

**Published:** 2019-05-24

**Authors:** Roman Szewczyk

**Affiliations:** Warsaw University of Technology, Institute of Metrology and Biomedical Engineering, Warsaw 02-525, Poland; szewczyk@mchtr.pw.edu.pl; Tel.: +48-609-464741

**Keywords:** magnetostriction model, inertial element, Mn–Zn ferrites

## Abstract

The present paper presents a new model of magnetostrictive hysteresis loop. A unified approach of both the hysteresis of *λ*(*B*) relation, as well as the lift-off phenomenon is proposed, which are explained together on the base of the response of the first order inertial element. Considering previously presented reports, the Maxwell–Boltzmann distribution based model of magnetostrictive characteristics with local maxima, enables modeling magnetostrictive loops. The model was validated on the results of measurements of magnetostrictive hysteresis loops of Mn_0.70_Zn_0_._24_Fe_2.06_O_4_ ferrite for power applications. Good agreement was confirmed for major magnetostrictive loop, especially for smaller values of flux density. As a result, the proposed model may be used for modeling the magnetostrictive response of inductive components of electrical machines, power conversion devices or magnetostrictive actuators.

## 1. Introduction

The magnetostriction phenomenon, observed for the first time by Joule in 1847 [1], is connected with changes of linear dimensions of the sample during the magnetization process. In spite of over hundred years of research on magnetostriction, it seems to still be one of the most mysterious macroscopic effects in the area of solid-state physics.

Magnetostriction is one of the sets of thermodynamically connected magneto-mechanical effects [2] summarized in Table 1.

Considering the fact, that the thermodynamically inverse effect to magnetostriction is the magnetoelastic Villari effect [16,17], it should be assumed, that magnetostriction is rather connected with changes of the magnetic state of material (described by its magnetization *M* or flux density *B*), than the value of external magnetizing field *H*. As a result, the magnetostriction *λ* compared to the magnetization *M* (or flux density *B*) dependence is the key for understanding the quantitative description of the magnetostrictive phenomenon.

Both the magnetostrictive hysteresis and lift-off phenomenon are very important from the point of view of modeling the magnetostrictive characteristics. These phenomena determine the accuracy of magnetostrictive positioning devices as well as being important for the development of the sensors utilizing magnetostrictive effects. For this reason, both are in the area of interests of engineers and scientists involved in research of soft magnetic materials.

The most efficient way to model the hysteresis loop is to model the anhysteretic curve first and later add the hysteresis and lift-off phenomenon description. The lift-off phenomenon is connected with the fact that the value of magnetostriction *λ* never returns to its initial value observed after demagnetization. The approach with a separate description of anhysteretic dependence and hysteresis description was taken in the Jiles-Atherton model of magnetic hysteresis loop [18]. Moreover, the magnetostrictive anhysteretic *λ*(*B*) dependence was presented previously for both monotonous curves [3,19], as well as for the curve with local maximum [20]. On the other hand, the hysteresis and the lift-off effect in the magnetostrictive *λ*(*B*) dependence was described only by phenomenological dependences [21]. Such dependences represent quite well the shape of *λ*(*B*), however, they don’t create the possibility of physical explanation of hysteresis phenomenon.

This paper presents a new approach to the modeling the both magnetostrictive *λ*(*B*) hysteresis and the lift-off phenomenon. With the use of the first order inertial element, both the hysteresis of magnetostriction and lift-off phenomenon can be modeled together. Such approach seems to be judged from the physical point of view due to the fact that the origins of these both effects seem to be similar. As a result, the proposed model enables deeper understanding of the physical mechanisms behind the magnetostriction. Moreover, it can be used for the development of magnetostrictive effect-based sensors and actuators, utilized in the industry [22] and medicine [23]. To sum up, the main goal of the paper is to propose the unified model of both magnetostrictive hysteresis and lift-off phenomenon, which can be useful for development of sensors and actuators control systems (e.g., precise micropositioning systems) as well as optimization of the structure of such devices.

## 2. The Method of Measurements of Magnetostriction and Magnetic Hysteresis Loop is Soft Ferrites

During the investigation, the frame-shaped sample [4] made of Mn_0.70_Zn_0.24_Fe_2.06_O_4_ ferrite for power applications was used. The sample had dimensions of 70 × 22 × 15 mm. The magnetizing winding had 25 turns, whereas flux density *B* sensing winding had 50 turns. Measurements were carried out in room temperature with the magnetizing field frequency of 1 Hz.

**Figure 1:**
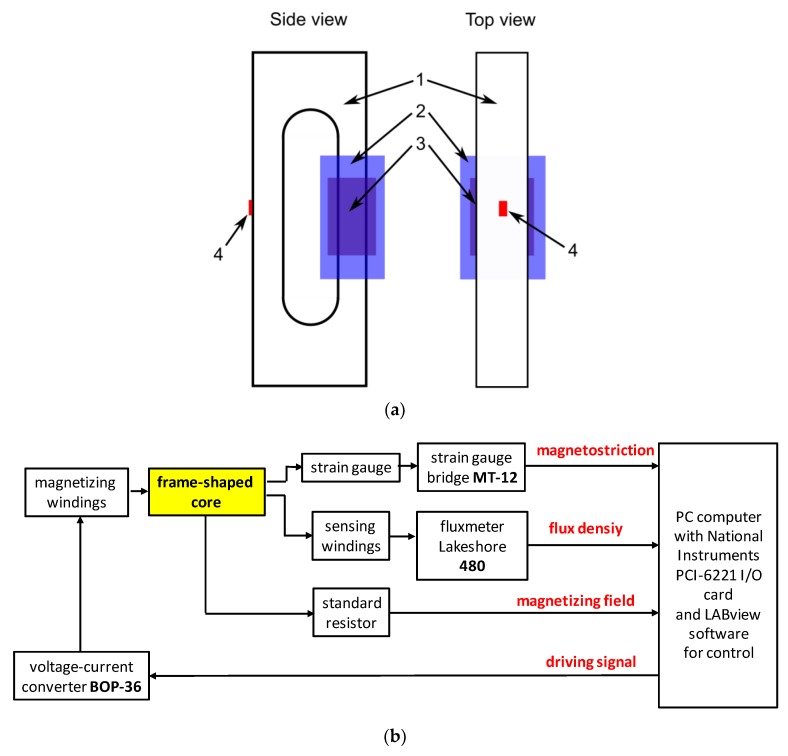
The method of investigation: (**a**) Schematic view of frame-shaped sample: 1—Tested sample, 2—magnetizing winding, 3—sensing winding, 4—semiconductor strain gauge; (**b**) computer controlled measurement stand enabling simultaneous measurements of magnetostriction *λ*, flux density *B* and magnetizing field *H*.

Measurements were carried out in the quasi-static mode. Due to the high resistivity of ferrites, eddy currents in the core are strongly limited. As a result, 1 Hz is suitable from the point of view of observations of both the magnetostrictive hysteresis and lift-off without the risk of time drift in the measurements of flux density *B*. The frame-shaped sample with a strain gauge is presented in Figure 1a, whereas the schematic block diagram of the experimental setup is presented in Figure 1b. The proposed, computer controlled measurement stand enabled simultaneous measurements of magnetostriction *λ*, flux density *B* and magnetizing field *H*. 

In the proposed measuring system, the magnetostriction is measured by the semiconductor strain gauge method [6]. For the measurements, AP120-6-12 semiconductor strain gauges (VTS, Zlin, Czech Republic) were used in connection with the MT-12 bridge (Meratronic, Warsaw, Poland). Flux density *B* is measured by the fluxmeter type 480 (Lakeshore, Carson, CA, USA), whereas the magnetizing field H is calculated from the electric current in magnetizing winding measured on the 1 Ω standard resistor. 

**Figure 2:**
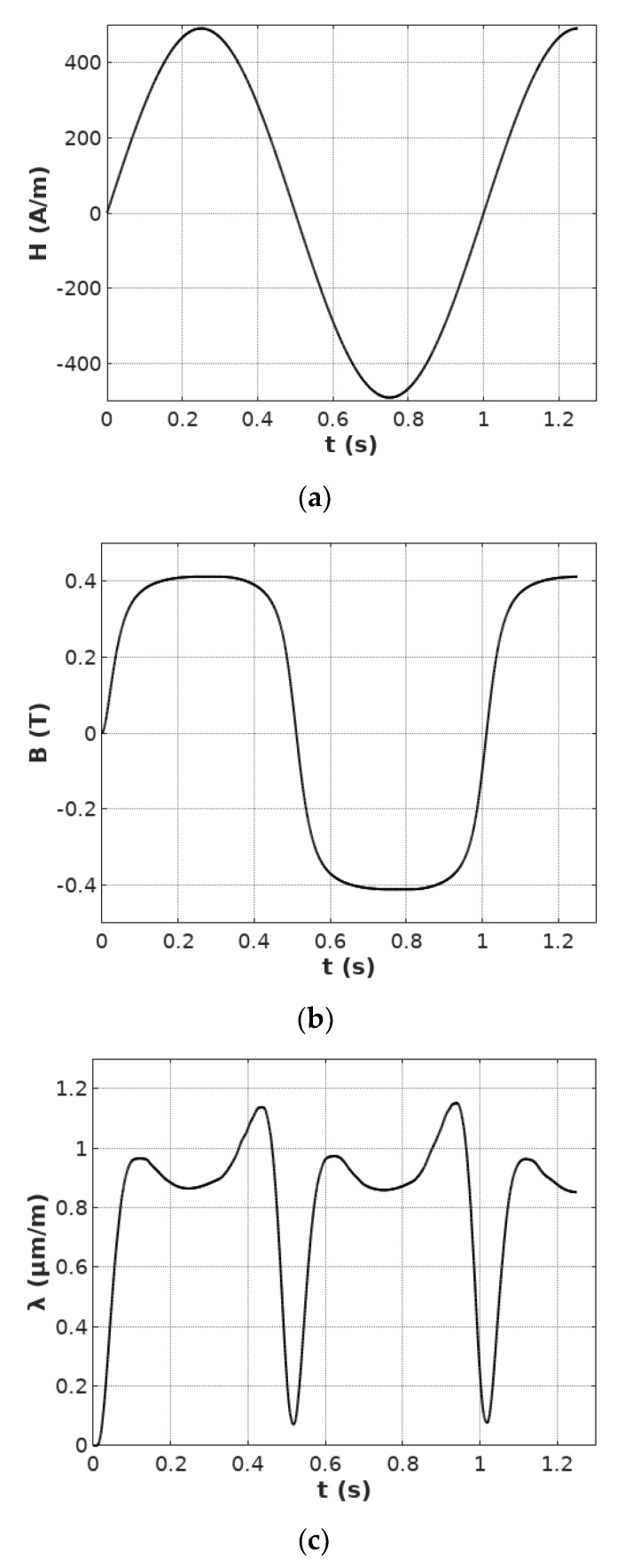
The results of measurements during the single measurement cycle: (**a**) Magnetizing field *H*, (**b**) flux density *B*, (**c**) magnetostriction *λ*.

Figure 2 presents the results of measurements of magnetizing field *H*, flux density *B* and magnetizing field *H* during the single measurement cycle.

**Figure 3:**
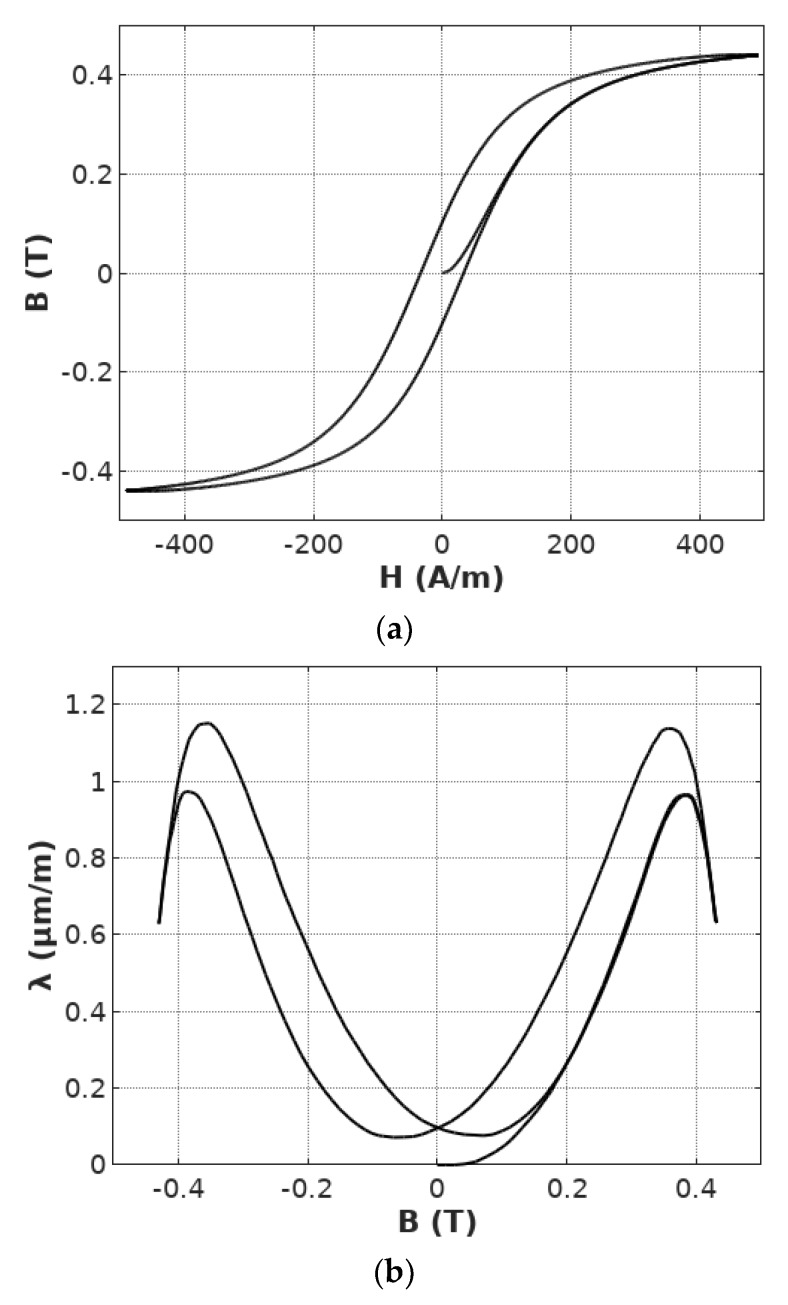
Results of measurements of hysteresis loops of Mn_0.70_Zn0._24_Fe_2.06_O_4_ ferrite for power applications: (**a**) *B*(*H*) magnetic hysteresis loop; (**b**) *λ*(*B*) magnetostrictive hysteresis loop (both magnetostrictive hysteresis and the lift-off phenomenon clearly visible).

On the basis of the results of measurements, both the *B*(*H*) magnetic hysteresis loop as well as *λ*(*B*) magnetostrictive hysteresis loop were calculated. Both these loops are presented in Figure 3. Magnetostrictive hysteresis as well as the lift-off phenomenon are clearly visible in Figure 3b.

## 3. Principles of Modeling the Anhysteretic Magnetostrictive Hysteresis Curve

Recently developed models of the anhysteretic magnetostrictive hysteresis curve utilized second [21] and fourth [24] order polynomials. The more sophisticated models are based on the differential equations considering both magnetostriction *λ_mov_*(*B*) caused the domain wall movement and magnetostriction *λ_rot_*(*B*) connected with the domain magnetization rotation. As a result, the differential equation describing anhysteretic *λ_anhyst_*(*B*) dependence is given by the following differential equations [20]:

(1) $$\frac{d\lambda_{mov}}{dB}=2a_{1}B$$

(2) $$\frac{d\lambda_{rot}}{dB}=a_{2}$$

(3) $$\frac{d\lambda_{anhyst}}{dB}=\frac{d\lambda_{mov}}{dB}\left(1-W\left(B\right)\right)+\frac{d\lambda_{rot}}{dB}W\left(B\right)$$

where *a*_1_ and *a*_2_ are parameters describing the slopes of the anhysteretic curve [20]. The initial condition *λ_anhyst_*(*B*) = 0 for *B* = 0.

Function *W*(*B*) determining the change of magnetization mechanism from the domain wall movement to the domain magnetization rotation is proposed in accordance to the Maxwell–Boltzmann statistical distribution given by the following equation [25]:

(4) $$W\left(B\right)=erf\left(\frac{B-B_{switch}}{k\sqrt{2}}\right)-\sqrt{\frac{2}{\pi }}\frac{\left(B-B_{switch}\right)e^{-\left(B-B_{switch}\right)/\left(2k^{2}\right)}}{k}$$

where *erf*() is the error function in to the Maxwell–Boltzmann statistical distribution [25]. Parameters *B_switch_* and *k* determines the change in magnetization mechanism from the domain wall to the domain magnetization rotation. The Maxwell–Boltzmann distribution was chosen due to the fact, that it is especially useful for the description of transition between different states in population, such as the transition between the magnetization mechanisms based on the domain wall movement and domain magnetization rotation in the population of domains.

**Figure 4:**
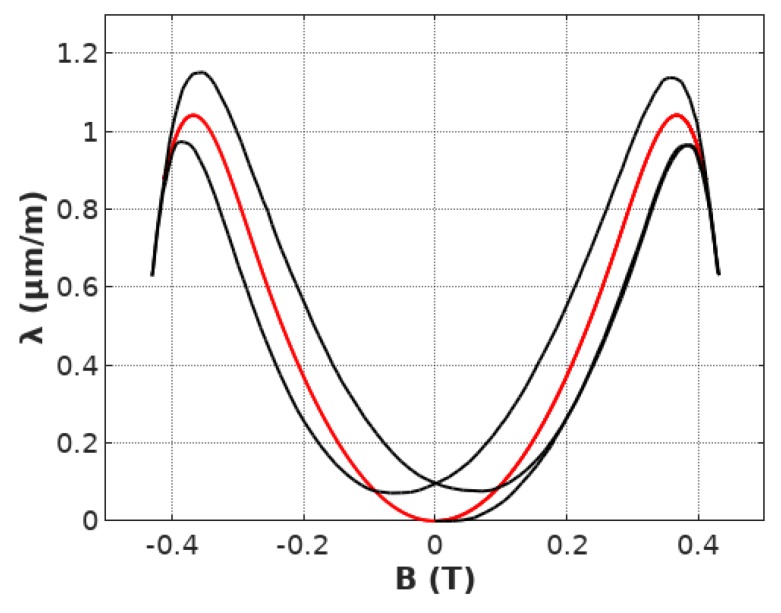
The results of fitting the magnetostrictive anhysteretic curve *λ_anhyst_*(*B*) to the results of experimental measurements of *λ*(*B*) dependence. Identified parameters: *a*_1_ = 9.3 $\frac{\mu m}{m}\frac{1}{T^{2}}$, *a*_2_ = −90.9 $\frac{\mu m}{m}\frac{1}{T}$, *B_switch_* = 0.248 T, *k* = 0.178.

Figure 4 presents the results of fitting the magnetostrictive anhysteretic curve *λ_anhyst_*(*B*) stated by Equations (1)–(4) into the results of experimental measurements. The parameter identification was carried out on the basis of the differential evolution algorithm with the target function *F* equal to the sum of the squared differences between the results of measurements and modeling [26].

## 4. Proposed, First Order Inertial Element Based Model of Magnetostrictive Hysteresis and Lift-Off Phenomenon

Inertial elements are the most common elements of linear automation control theory. The most common example of the first order inertial element is the resistor-capacitor circuit [27]. The first order inertial element can be described by the Laplace transform equation:

(5) $$ℒ\left(s\right)=\frac{1}{Ts+1}$$

where *T* is the time constant of the first order inertial element.

Bode diagrams presenting amplitude and phase shift dependences of the first order inertial element are widely presented in the literature [28]. The cut-off frequency *f_c_* of such element is equal:

(6) $$f_{c}=\frac{1}{T}$$

For the numerical calculation, the first order inertial element may be represented by the first order, low pass Butterworth filter [29]. In the case of such filter, the *a_cut-off_* filter parameter is given as:

(7) $$a_{cut-off}=\frac{2f_{c}}{f_{s}}$$

where *f_s_* is the sampling frequency. Let’s assume, that time constant *T* of the inertial element is given as:

(8) $$T=a_{inert}\cdot T_{s}$$

where *T_s_* = 1/*f_s_* is the time between samples of magnetostriction signal and *a_inert_* is the dimensionless constant. In such a case, considering Equation (6):

(9) $$f_{c}=\frac{1}{a_{inert}T_{s}}=\frac{f_{s}}{a_{inert}}\;$$

After the re-arrangement and considering Equation (7), we get dimension less parameter *a_cut-off_* equal:

(10) $$a_{cut-off}=\frac{2}{a_{inert}}$$

**Figure 5:**
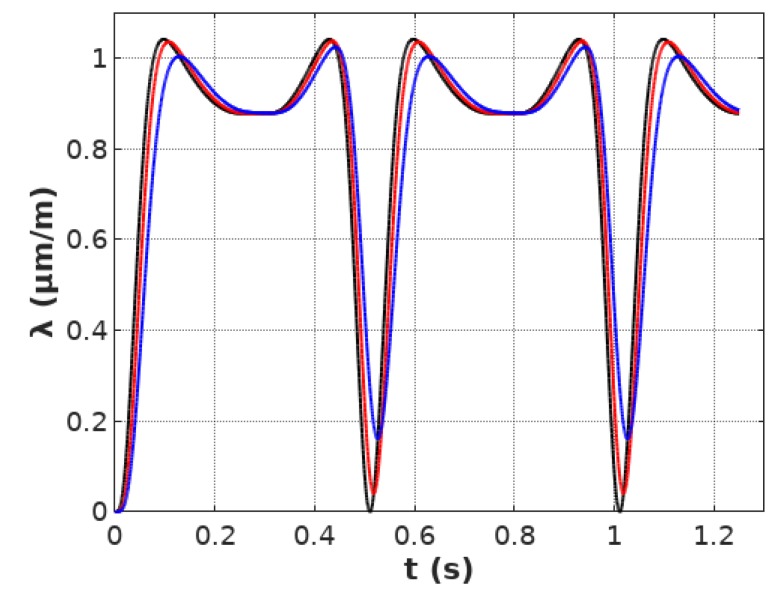
Magnetostriction *λ*(*t*) signal calculated for parameters: *a*_1_ = 9.3 $\frac{\mu m}{m}\frac{1}{T^{2}}$, *a*_2_ = −90.9 $\frac{\mu m}{m}\frac{1}{T}$, *B_switch_* = 0.248 T, *k* = 0.178, filtered by the first order Butterworth filter for *a_inert_* equal: 2 (black), 80 (red), 200 (blue).

Figure 5 presents the magnetostriction *λ*(*t*) signal calculated from Equation (3) and curve presented in Figure 2b, considering parameters: *a*_1_ *=* 9.3 $\frac{\mu m}{m}\frac{1}{T^{2}}$, *a*_2_ *=* −90.9 $\frac{\mu m}{m}\frac{1}{T}$, *B_switch_ =* 0.248 T, *k =* 0.178, filtered by the first order Butterworth filter for different values of *a_inert_* parameters. 

**Figure 6:**
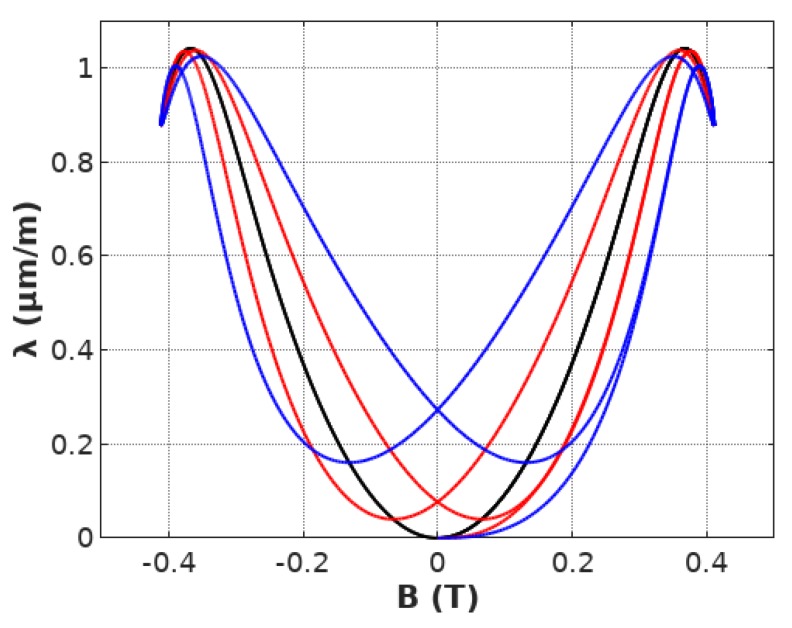
Magnetostriction *λ*(*B*) hysteresis loop calculated for parameters: *a*_1_ = 9.3 $\frac{\mu m}{m}\frac{1}{T^{2}}$, *a_2_* = −90.9 $\frac{\mu m}{m}\frac{1}{T}$, *B_switch_* = 0.248 T, *k* = 0.178, filtered by the first order Butterworth filter for *a_inert_* equal: 2 (black), 80 (red), 200 (blue).

It can be observed, that the introduction of the first order inertial element (represented by the first order Butterworth filter) leads to lift-off *λ*(*t*) of the signal as well as to its phase shift. The results of such filtering on *λ*(*B*) characteristics can be observed in Figure 6.

Moreover, increasing the time constant *T* of the first order inertial element leads to both the increase of hysteresis on *λ*(*B*) dependence as well as the increase of the lift-off effect. Such observation indicates, that both of these phenomena may have similar origins connected probably with energy dissipation and resistance during the magnetic domain wall movement.

The proposed model distinguishes the anhysteretic magnetization curve and hysteresis similarly to the approach proposed for the magnetic hysteresis loop in the Jiles-Atherton model [18]. However, it considers a different approach, than the extension to this model proposed by Sablik et al. [7]. It should be highlighted that the proposed model enables consideration of magnetostrictive hysteresis and the lift-off phenomenon jointly, as the different phenomena with the same origins.

## 5. Validation of the Model 

**Figure 7:**
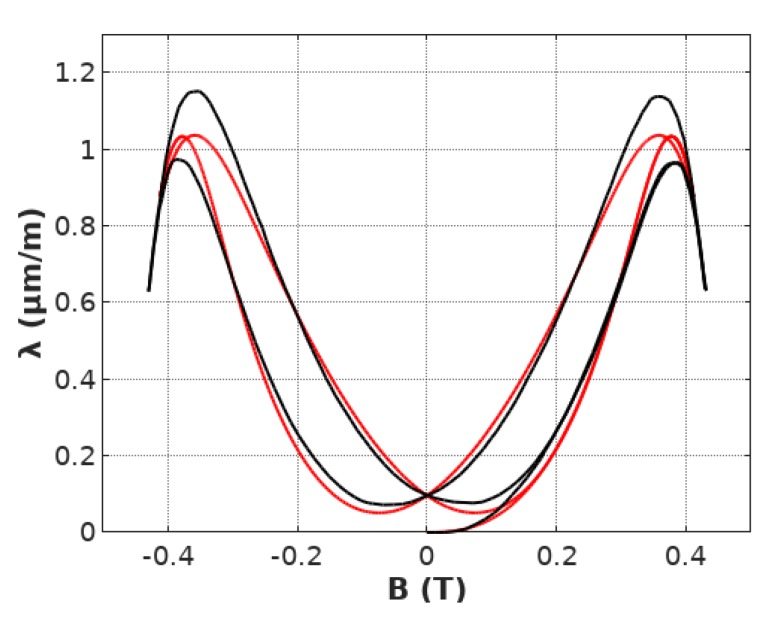
Magnetostrictive *B*(*H*) hysteresis loop of Mn_0.70_Zn0._24_Fe_2.06_O_4_ ferrite for power application modeled by the first order inertial element, calculated for the parameters: *a*_1_ *=* 9.3 $\frac{\mu m}{m}\frac{1}{T^{2}}$*, a*_2_ *=* −90.9 $\frac{\mu m}{m}\frac{1}{T}$ *, B_switch_ =* 0.248 *T, k =* 0.178 (experimental measurements—black line, results of modeling—red line).

The proposed model of hysteresis and lift-off phenomenon in the magnetostrictive *λ*(*B*) hysteresis loop was implemented in Octave 4.4.1, open-source MATLAB alternative. Anhysteretic loop model parameters were identified previously: *a*_1_ = 9.3 $\frac{\mu m}{m}\frac{1}{T^{2}}$, *a*_2_ = −90.9 $\frac{\mu m}{m}\frac{1}{T}$, *B_switch_* = 0.248 T, *k* = 0.178. The value of parameter *a_inert_* equal to 91.89 was identified by the Nedler-Mead optimization algorithm [30] with the same target function *F* equal to the sum of the squared differences between the results of measurements and modeling. The final results of modeling the hysteresis and lift-off are presented in Figure 7.

It can be observed, that the proposed, first order inertial element-based model very well describes the shape of magnetostrictive *λ*(*B*) hysteresis loop for smaller values of flux density *B*. For these areas of magnetostrictive *λ*(*B*) hysteresis loop both the hysteresis and lift-off phenomenon are very well represented. However, more significant differences between experimental results and results of modeling occur for the area of transition of magnetization mechanism between the domain wall movement and domain magnetization rotations. In the presented case, this transformation occurs roughly for flux density *B* between 0.3 and 0.4 T. For this range of flux density B differences are the most significant. It should be highlighted, that these differences indicate, that this transition should be the subject of further research connected with its physical principles and sources of hysteresis in this part of the magnetostrictive *λ*(*B*) hysteresis loop.

Presented results are in line with the observation, that in the case of ceramic soft magnetic materials, such as Mn–Zn ferrites, the domain wall movement is the significant part of the magnetization process. The magnetization rotation occurs only for higher values of flux density. Moreover, the domain rotation is not connected with the significant hysteresis (both magnetic and magnetostrictive) due to small values of monocrystalline anisotropy energy.

## 6. Conclusions

The results of modeling presented in the paper clearly confirms that magnetostrictive hysteresis and the lift-off phenomenon observed in the *λ*(*B*) loop can be jointly described by the proposed model based on the first order inertia element. As a result, magnetostrictive *λ*(*B*) hysteresis loop can be described by the set of five parameters, where only one parameter is connected with the hysteresis and lift-off phenomenon.

The proposed model very well represents the magnetostrictive hysteresis *λ*(*B*) loop for smaller values of flux density *B*. However, the differences occur for the area of transition of magnetization mechanism between the domain wall movement and domain magnetization rotations. These differences confirm the significance and necessity of further research on physical mechanisms behind this transition.

In addition, the proposed model can be especially useful for technical applications. Typically, Mn_0.70_Zn0._24_Fe_2.06_O_4_ ferrites for power application operate in the area of initial values of flux density *B*. As a result, the proposed model will very well describe the magnetostrictive hysteresis loops enabling optimization of magneto-mechanical properties of power conversion devices with inductive components with cores made of soft ferrites. Moreover, the proposed unified model of both magnetostrictive hysteresis and the lift-off phenomenon can be useful during the process of development of sensors and actuators control systems, especially precise micropositioning systems.

It should be also highlighted, that the application of the proposed model is not limited to Mn–Zn ferrites. Due to the similar phenomena occurring during the magnetization process as well as the similar character of magnetostrictive hysteresis loop, it can also be efficiently used for modeling the magnetostrictive characteristics of amorphous [31] and nanocrystalline ribbons [32].

## Figures and Tables

**Table 1:** Thermodynamic connections among magneto-mechanical effects [3].

Effect	Thermodynamically Inverse Effect
**Magnetostrictive effect** Changes of the sample linear dimensions during its magnetization process [4,5]	**Villari effect** Changes of the sample magnetization under the compressive or tensile stresses [6,7]
**Wiedemann effect** Twisting of the magnetic wire with electric current under the longitudinal magnetic field [8,9]	**Inverse Wiedemann effect** Magnetization of the wire due to the twisting, in the presence of circular anisotropy [10,11]
**Barrett effect** Changes of the sample volume during its magnetization process [12,13]	**Nagaoka-Honda effect** Changes of the sample magnetization under the hydrostatic pressure [14,15]
